# Perturbations of pulsatile hemodynamics and clinical outcomes in patients with acute heart failure and reduced, mid-range or preserved ejection fraction

**DOI:** 10.1371/journal.pone.0220183

**Published:** 2019-08-05

**Authors:** Wei-Ming Huang, Shih-Hsien Sung, Wen-Chung Yu, Hao-Min Cheng, Chi-Jung Huang, Chao-Yu Guo, Dai-Yin Lu, Ching-Wei Lee, Chen-Huan Chen

**Affiliations:** 1 Department of Medicine, Taipei Veterans General Hospital, Taipei, Taiwan; 2 Department of Medicine, National Yang-Ming University, Taipei, Taiwan; 3 Department of Public Health, National Yang-Ming University, Taipei, Taiwan; 4 Department of Medical Education, Taipei Veterans General Hospital, Taipei, Taiwan; Temple University, UNITED STATES

## Abstract

**Background:**

Heart failure with mid-range ejection fraction (HFmrEF) has been proposed as a new phenotype of heart failure. We therefore investigated the pulsatile hemodynamic characteristics and outcomes in patients with HFmrEF, in comparison with those with reduced (HFrEF) or preserved (HFpEF) ejection fraction.

**Methods:**

The study was composed of two cohorts of patients hospitalized due to acute heart failure. Pulsatile hemodynamic measures, including carotid-femoral pulse wave velocity (cf-PWV), carotid pulse pressure (cPP), amplitude of the backward pressure wave (Pb) and carotid augmentation index (cAIx), were recorded on admission and before discharge in Cohort A (n = 230, mean age 69.9 ±15.4 years), and long-term follow-up was performed in Cohort B (n = 2677, mean age 76.3 ± 33.4 years).

**Results:**

In Cohort A, patients with HFmrEF had persistently greater cf-PWV, cPP, Pb, and cAI than those with HFrEF, both on admission and before discharge. In contrast, patients with HFmrEF and HFpEF had similar pulsatile hemodynamic characteristics. In cohort B, patients with HFmrEF and HFrEF had similar three-year mortality rates and both were significantly higher than that in patients with HFpEF (both P values < 0.05).

**Conclusions:**

Patients with HFmrEF were characterized by a worse left ventricular systolic function than patients with HFpEF and excessive wave reflections than patients with HFrEF. Future studies are required to confirm that the unfavorable ventriculo-arterial coupling in HFmrEF might play a role in the pathogenesis of high long-term mortality in these patients.

## Introduction

A new phenotype of heart failure (HF) with mid-range ejection fraction (HFmrEF) is referred to HF patients with left ventricular ejection fraction (LVEF) of 40% to 49% [1]. HFmrEF represents a gray zone regarding evidence-based therapy while the majority of the clinical trials have enrolled HF patients with a LVEF of <40% (heart failure with reduced ejection fraction, HFrEF) or ≥50% (heart failure with preserved ejection fraction, HFpEF) [2–10]. Compared with HFrEF, HFpEF accounted for at least 50% of all hospital admissions for HF and had unique pressure-volume relationships. [11–13].

The clinical characteristics of HFmrEF were considered to be intermediate between those of HFrEF and HFpEF, regarding age and co-morbidities [12, 14–16]. In terms of clinical outcomes, Berry et al. have demonstrated in a meta-analysis of 50,991 subjects with chronic heart failure (CHF) that the risk of death increased notably and linearly once the LVEF fell below 40% [17]. For those with LVEF ≥40%, LVEF wasn’t related to mortality [17]. The results of MAGGIC study may suggest HFmrEF, as HFpEF has better clinical outcomes than HFrEF.

He et al. have reported a progressively downward and rightward shift of end-systolic or end-diastolic pressure-volume relations from patients with HFpEF, HFmrEF and HFrEF [12]. When the stroke volume was similar, the left ventricular end-diastolic volume increased along with the order of HFpEF, HFmrEF and HFrEF [12]. In addition, HFpEF has higher central blood pressures and excessive wave reflections, but comparable arterial stiffness as HFrEF [18]. However, the pulsatile hemodynamics of HFmrEF in the comparisons with the others need to be elucidated, when arterial stiffness and wave reflections have been related to adverse events of patients with acute heart failure (AHF) [19, 20]. In the present study, we therefore investigated the changes of arterial functions, the cardiac performance, and the prognosis of phenotypes of HF.

## Methods

### Study population

The study was composed of two cohorts of our previous work and an intramural registry of Taipei Veterans General Hospital of acute heart failure (AHF) [20, 21]. AHF was delimited as new‐onset or gradually or rapidly worsening heart failure symptoms and signs requiring hospitalizations [22]. Cohort A of AHF and sinus rhythm has been enrolled for a series measures of pulsatile hemodynamics [19, 20]. The written informed consents were obtained. Cohort B was derived from the registry, which was conducted to recruit AHF patients from October 2003 to December 2012 for the survey of AHF long-term outcomes [21, 23, 24]. Informed consent was waived in Cohort B by the ethics committee. The investigation conformed to the principles outlined in the Declaration of Helsinki. It was approved by the institutional review board of Taipei Veterans General Hospital.

### Pulsatile hemodynamics, echocardiogram and data collection

In cohort A, pulsatile hemodynamics was measured within 24 h of hospitalization and pre-discharge after resting for at least 10 minutes in a quiet, temperature-controlled room. Cardiac index, stroke volume, and systemic vascular resistance index (SVRI) were recorded by impedance cardiography (BioZ ICGMonitor, CardioDynamics, CA, USA) [19, 20]. Carotid–femoral pulse wave velocity (cf-PWV) was measured from the foot-to-foot pulse transit time and the traveling distance between the right carotid and right femoral arteries as our previous work [19, 20]. The carotid pressure waveform with its forward (Pf) and backward components (Pb) and carotid augmentation index (cAIx) was obtained by tonometry (VP-2000, Colin Corporation, Komaki, Japan) and pressure wave analysis [19, 20]. The intra- and interobserver intraclass correlation coefficients have been validated in our previous work [25].

Left ventricular ejection fraction (LVEF) was calculated from Simpson’s method [26] in cohort A and 2-D M-mode modified Ellipsoid method [27] in cohort B. Left ventricular internal dimension at diastolic and systolic (LVIDd and LVIDs) were recorded accordingly. The peak of early (E) and late (A) mitral inflow was obtained. The measures of tissue velocity (e’) at septal and lateral mitral annulus were determined by using tissue Doppler. Pulmonary artery systolic pressure (PASP) was also estimated. Eccentric hypertrophy was defined as a relative wall thickness (RWT) ≤0.42 and a posterior wall thickness >10mm. All the measures of cohort A were acquired and analyzed by S.H.S. Echocardiographic data of cohort B were acquired by four technicians and interpreted by S.H.S. and W.C.Y.

Data of demographic characteristics, hemogram, and biochemistry were collected from a web-based electronic medical recording system. Estimated glomerular filtration rate (eGFR) was determined by the modified glomerular filtration rate estimating equation for Chinese patients [28]. Because the commercialized measure for N-terminal pro-brain natriuretic peptide (NT-proBNP; Roche Diagnostics, Basel, Switzerland) was available after 2009, there were missing values of NT-proBNP in cohort B.

### Follow-up

Cohort A was followed by clinical visits, telephone contacts and review of medical records for a year. Major adverse cardiovascular events (MACEs) were referred to death, myocardial infarction, stroke and hospitalization for HF. In Cohort B, the date and causes of death of participants were obtained by linking our registry with the National Death Registry. [29].

### Statistical analysis

Continuous variables were presented as mean ± standard deviation. Categorical variables were expressed as the absolute numbers and relative frequencies. Student's t-test or Chi-square tests were calculated for the baseline characteristics comparisons where appropriate. The changes of pulsatile hemodynamics during hospitalization were evaluated by paired-t test. Kaplan-Meier survival curve analysis demonstrated the outcomes of the 3 phenotypes of HF. Cox proportional hazards models were used to determine the pulsatile hemodynamics in the prediction of MACEs. All the statistical analyses were performed SPSS v.20.0 software (SPSS, Inc., Chicago, IL, USA) and the performed tests were two-sided. A P value < 0.05 was considered statistically significant.

### Results

A total of 230 patients (age 69.9 ± 15.4 years, 77% men) in ***Cohort A*** were analyzed, and the baseline characteristics of HFrEF, HFmrEF and HFpEF were demonstrated in Table 1. Patients with HFpEF were the oldest and most prevalent with hypertension. The distribution of gender, diabetes, coronary artery disease and dyslipidemia were similar between groups. LVEF increased, and LVIDd and LVIDs decreased along with the order of HFrEF, HFmrEF and HFpEF. HFpEF had the lowest septal E/e’ and the least prevalence of eccentric hypertrophy, while HFrEF had the largest left atrial diameter. In addition, HFrEF had the highest hemoglobin and NT-proBNP levels. While renin-angiotensin system (RAS) inhibitors were prescribed equally in the three groups, both HFrEF and HFmrEF would receive more prescription of β-blockers than HFpEF. In addition, patients with HFrEF were more likely to take minerocorticoid antagonist and digoxin.

**Table 1:** Baseline characteristics of the Cohort A.

	HFrEF (n = 138)	HFmrEF (n = 36)	HFpEF (n = 56)	P value
***Age (years)***	67.2 ± 15.8	71.5 ± 16.2	78.3 ± 9.7^†^	0.013
***Male gender*, *n (%)***	109 (79.6)	26 (72.2)	44 (78.6)	0.635
***De novo heart failure***	81 (58.7)	19 (52.9)	31 (55.4)	0.785
***Smoker***	51 (37.0)	11 (30.6)	20 (35.7)	0.775
***Co-morbidity*, *n (%)***				
Hypertension	92 (67.2)	28 (77.8)	48 (85.7)^†^	0.024
Diabetes mellitus	59 (43.1)	23 (63.9)	27 (48.2)	0.083
Coronary artery disease	86 (62.3)	21 (58.3)	26 (46.4)	0.127
Dyslipidemia	38 (28.1)	7 (19.4)	12 (22.2)	0.472
***Echocardiography***				
LVEF (%)	27.3 ± 6.9^Ŧ^	44.8 ± 2.8^†^	59.2 ± 7.2^†^^Ŧ^	<0.001
Septal E/e’	20.9 ± 10.7	19.2 ± 7.9	14.3 ± 7.3^†^	<0.001
LA diameter (mm)	42.2 ± 5.9	39.5 ± 7.7	40.1 ± 6.4	0.028
LVIDd (mm)	63.0 ± 9.5^Ŧ^	56.9 ± 8.2^†^	51.2 ± 8.1^†^^Ŧ^	<0.001
LVIDs (mm)	53.1 ± 9.5^Ŧ^	42.2 ± 7.9^†^	34.3 ± 7.3^†^^Ŧ^	<0.001
Eccentric hypertrophy, *n (%)*	47 (34.1)	13 (36.1)	9 (16.1)^†^	0.032
PASP (mmHg)	44.3 ± 15.7	44.3 ± 18.5	43.1 ± 15.6	0.911
***Hemogram and Biochemistry*, *on Admission***				
Hemoglobin (g/dl)	12.4 ± 2.2	11.0 ± 2.1^†^	11.6 ± 2.1	0.004
eGFR (mL/min/1.73m^2^)	54.3 ± 28.9	49.1 ± 30.6	50.9 ± 22.7	0.540
Sodium (mEq/L)	138.1 ± 4.1	138.6 ± 5.1	138.4 ± 5.3	0.805
Potassium (mEq/L)	4.08 ± 0.68	4.12 ± 0.56	4.07 ± 0.64	0.932
^$^ Ln NT-proBNP (pg/ml)	8.15 ± 1.34	7.68 ± 1.74	6.73 ± 1.54^†^^Ŧ^	<0.001
***Medications*, *n (%)***				
Beta-blocker	91 (67.4)	28 (77.8)	21 (38.9)^†^^Ŧ^	<0.001
RAS inhibitors	100 (72.5)	26 (72.2)	40 (71.4)	0.989
Spironolactone	89 (65.9)	19 (52.8)	24 (44.4)^†^	<0.001
Digoxin	38 (28.1)	3 (8.3)^†^	3 (5.6)^†^	<0.001

$ Geometric means and standard deviation

† indicated significant P values of < 0.05, compared with HFrEF in post-hoc analysis

Ŧ indicated significant P values of < 0.05, compared with HFmrEF in post-hoc analysis

e’: early diastolic tissue velocity mitral annulus; E/e’: ratio of early ventricular filling velocity (E) to early diastolic tissue velocity mitral annulus; EF: ejection fraction; eGFR: estimated glomerular filtration rate; LA diameter: the diameter of left atrium; LV: left ventricular

LVIDd: left ventricular internal diameter at end diastole; LVIDs: left ventricular internal diameter at end systole; MACE: major adverse cardiac events, including re-hospitalization for heart failure, non-fatal myocardial in-farction, non-fatal stroke, and death; NT-proBNP: N-terminal pro-brain natriuretic peptide; PASP: pulmonary artery systolic pressure; RAS inhibitors: renin-angiotensin system inhibitors

In ***Cohort B*** of 2677 patients (age 76.3 ± 33.4, 67% men), HFpEF was the oldest and most likely to be women. (Table 2) De novo HF was higher in HFrEF than the others. Prevalence of hypertension was again highest in patients with HFpEF, while diabetes and coronary artery disease were less present in patients with HFrEF and HFpEF. In addition, values of LVEF, LVIDd, LVIDs and Septal E/e’ in HFmrEF significantly lay between HFrEF and HFpEF. However, eccentric LVH was less present in HFpEF while LA diameter was similar between groups. While hemoglobin levels increased along with the order of HFpEF, HFmrEF and HFrEF, NT-proBNP was lower in HFpEF and eGFR was not different.

**Table 2:** Baseline characteristics of Cohort B.

	HFrEF (n = 690)	HFmrEF (n = 372)	HFpEF (n = 1615)	P value
***Age (years)***	67.2 ± 15.8^Ŧ^	71.5 ± 16.2^†^	78.3 ± 9.7^†^^Ŧ^	<0.001
***Male gender*, *n (%)***	535 (77.5)^Ŧ^	260 (69.9)^†^	992 (61.5)^†^^Ŧ^	<0.001
***De novo heart failure***	200 (29.2)	70 (18.9) ^†^	309 (19.1) ^†^	<0.001
***Co-morbidity*, *n (%)***				
Hypertension	347 (50.3)	214 (57.5)	1077 (66.7)^†^^Ŧ^	<0.001
Diabetes mellitus	217 (31.4)	148 (39.8)^†^	634 (39.3)^†^	0.001
Coronary artery disease	305 (44.2)	153 (41.1)	493 (30.5)^†^^Ŧ^	<0.001
Atrial fibrillation	200 (29.0)	107 (28.8)	479 (29.7)	0.914
Dyslipidemia	68 (9.9)	34 (9.1)	168 (10.4)	0.746
***Echocardiography***				
LVEF (%)	28.3 ± 15.0^Ŧ^	45.1 ± 2.9^†^	67.4 ± 10.3^†^^Ŧ^	<0.001
Septal E/e’	20.8 ± 8.9^Ŧ^	18.6 ± 8.4^†^	16.7 ± 7.2^†^^Ŧ^	<0.001
LA diameter (mm)	45.8 ± 8.0	45.7 ± 9.0	45.6 ± 9.1	0.890
LVIDd (mm)	61.4 ± 10.2^Ŧ^	57.4 ± 8.9^†^	50.6 ± 8.4^†^^Ŧ^	<0.001
LVIDs (mm)	52.9 ± 9.0^Ŧ^	44.3 ± 6.9^†^	31.4 ± 7.6^†^^Ŧ^	<0.001
Eccentric hypertrophy, *n (%)*	175 (25.4)	90 (24.2)	227 (14.1)^†^^Ŧ^	<0.001
PASP (mmHg)	45.7 ± 16.7	44.0 ± 15.9	43.3 ± 16.5^†^	0.011
***Hemogram and Biochemistry*, *on Admission***				
Hemoglobin (g/dl)	12.6 ± 2.1^Ŧ^	11.8 ± 2.2^†^	11.4± 2.1^†^^Ŧ^	<0.001
eGFR (mL/min/1.73m^2^)	54.8 ± 27.4	52.9 ± 30.1	52.0 ± 31.4	0.126
Sodium (mEq/L)	138.8 ± 4.4	139.1 ± 4.0	138.7 ± 4.9	0.421
Potassium (mEq/L)	4.11 ± 0.69	4.03 ± 0.62	4.12 ± 0.70	0.097
^$^ Ln NT-proBNP (pg/ml), n = 1027	8.97 ± 1.23	8.97 ± 0.97	8.34 ± 1.43^†^^Ŧ^	<0.001
***Medications*, *n (%)***				
Beta-blocker	484 (70.1)	255 (68.5)	968 (59.9)^†^^Ŧ^	<0.001
RAS inhibitors	579 (83.9)	323 (86.8)	1341 (83.0)	0.200
Spironolactone	481 (69.7)	234 (62.9)	838 (51.9)^†^^Ŧ^	<0.001
Digoxin	303 (43.9)	228 (38.7)	471 (29.2)^†^^Ŧ^	<0.001

$ Geometric means and standard deviation

† indicated significant P values of < 0.05, compared with HFrEF in post-hoc analysis

Ŧ indicated significant P values of < 0.05, compared with HFmrEF in post-hoc analysis

e’: early diastolic tissue velocity mitral annulus; E/e’: ratio of early ventricular filling velocity (E) to early diastolic tissue velocity mitral annulus; EF: ejection fraction; eGFR: estimated glomerular filtration rate; LA diameter: the diameter of left atrium; LV: left ventricular

LVIDd: left ventricular internal diameter at end diastole; LVIDs: left ventricular internal diameter at end systole; NT-proBNP: N-terminal pro-brain natriuretic peptide; PASP: pulmonary artery systolic pressure; RAS inhibitors: renin-angiotensin system inhibitors

### Pulsatile hemodynamics during hospitalization

**Fig 1:**
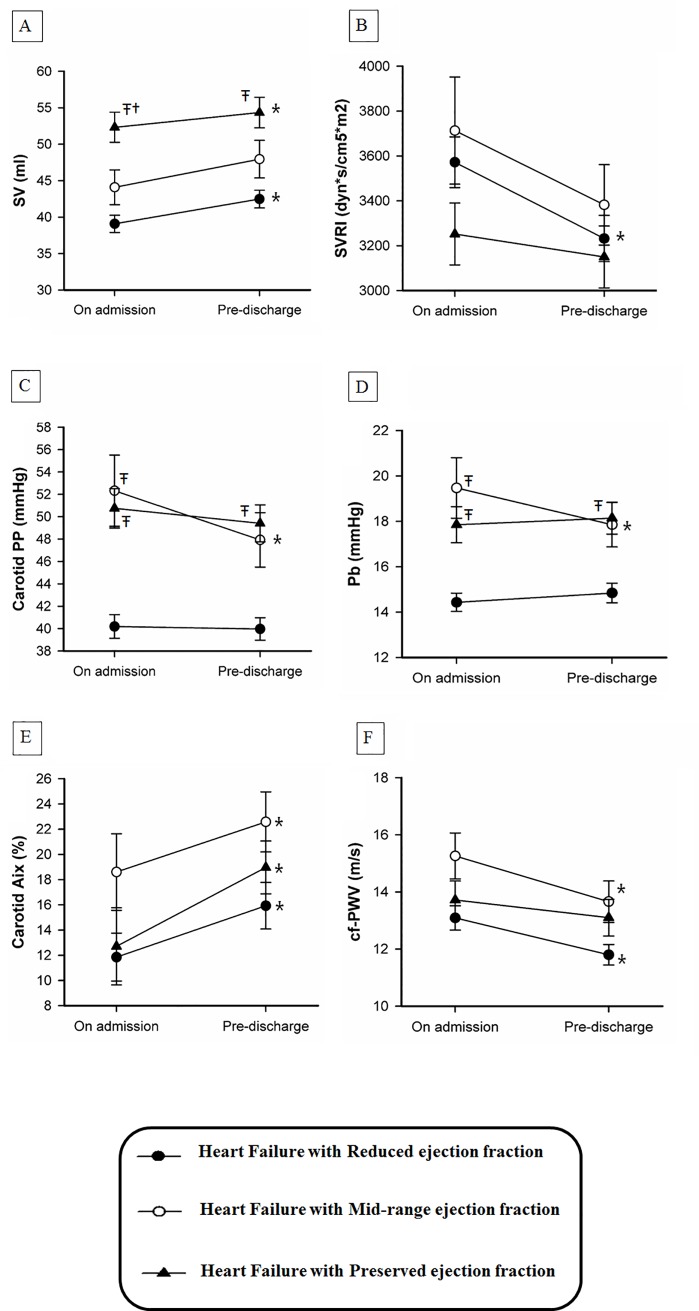
Mean ± Standard Error of measure of (A) stroke volume, (B) systemic vascular resistance index (SVRI), (C) carotid pulse pressure (carotid PP), (D) reflected wave amplitude (Pb), (E) carotid augmentation index (cAIx), and (F) carotid-femoral pulse wave velocity (cf-PWV) during the hospitalizations, stratified by the phenotypes of heart failure. † indicated a P value of < 0.05, compared with HFrEF in post-hoc analysis; ^Ŧ^ indicated a P values of < 0.05, compared with HFmrEF in post-hoc analysis; * indicated a P values of < 0.05 for the changes of the hemodynamic indices during the hospitalizations using paired-t test.

The hemodynamic changes of Cohort A during the index hospitalization have been demonstrated in Fig 1. In short, HFpEF has the highest stroke volume (SV) on admission and at discharge among the study population, while both HFpEF and HFrEF would experience a significant improvement in stroke volume after treatment. (Fig 1A) In addition, the three phenotypic HF have similar levels of on-admission and pre-discharge SVRI. Only HFrEF would experience a significant reduction of SVRI. (Fig 1B) Both HFpEF and HFmrEF have higher carotid pulse pressure (cPP) and Pb than HFrEF on admission, and HFmrEF would have a significant reduction of cPP and Pb at discharge. (Fig 1C and 1D) The on-admission and pre-discharge cf-PWV and cAIx were not different between groups, however, all of them would have increased cAIx and decreased cf-PWV during the hospitalizations. (Fig 1E and 1F)

### Mortality of the three phenotypic heart failure

**Fig 2:**
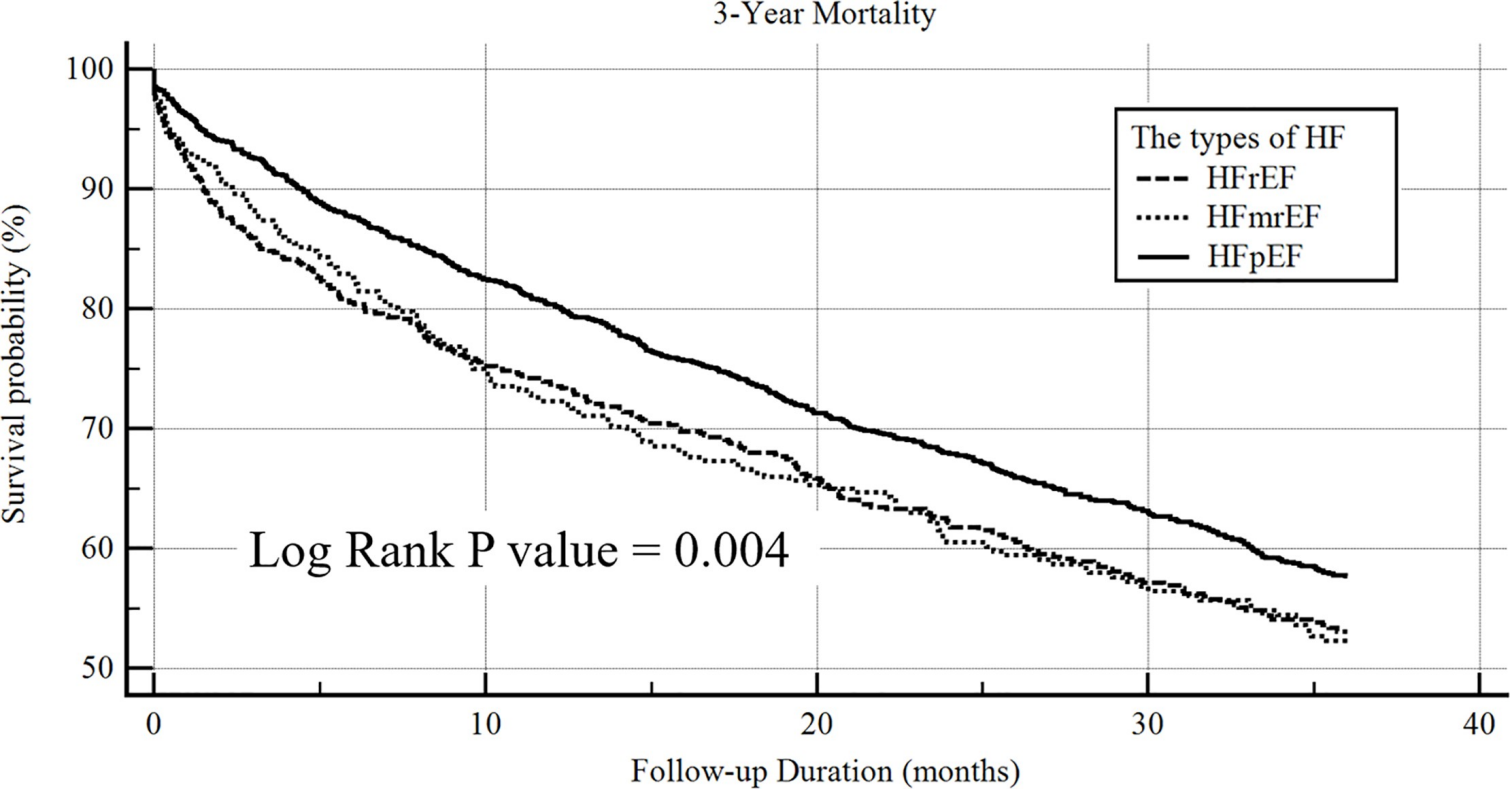
The Kaplan–Meier survival curve analysis of the study population, stratified by the phenotypes of heart failure for 3-year all-cause mortality.

Among 2677 subjects of Cohort B, 1004 patients died during a mean follow-up duration of 21.3±13.6 months. The Kaplan-Meier survival curve analyses showed that both HFrEF and HFmrEF shared the similarly higher risks of mortality than HFpEF. (Fig 2) With adjustments for age, sex, eGFR, and hemoglobin levels, both HFrEF and HFmrEF remained carried higher risks of mortality [hazard ration and 95% confidence interval, referent to HFpEF: 1.753 (1.488–2.065) and 1.474 (1.211–1.794), respectively].

### Predictors of major adverse cardiac events in phenotypic heart failure

In cohort A of 230 subjects that 62 patients died and 105 patients experienced MACEs during a mean follow-up duration of 10.2 ± 3.5 months. In this particular cohort, we did not observe the survival difference between the three phenotypic HF. However, carotid PP and Pb were significantly associated with 1-year MACEs in patients with HFrEF. In contrast, cf-PWV was related to the outcomes of patients with HFpEF. In HFmrEF, only Pb was related to post-discharge adverse events. (Table 3)

**Table 3:** The predictors value of pre-discharge pulsatile hemodynamics of 1-year MACE identified by multivariate variate Cox regression analysis.

	HFrEF		HFmrEF		HFpEF	
	HR (95% CI)	P value	HR (95% CI)	P value	HR (95% CI)	P value
**cPP (mmHg)**	1.041 (1.001–1.081)	0.042	1.138 (0.370–3.501)	0.822	1.034 (1.000–1.070)	0.052
**cf-PWV (m/s)**	1.063 (1.000–1.131)	0.050	1.013 (0.902–1.139)	0.823	1.136 (1.037–1.246)	0.006
**cAIx (%)**	1.015 (1.000–1.030)	0.051	1.033 (0.993–1.074)	0.103	1.010 (0.985–1.036)	0.423
**Pb (mmHg)**	1.091 (1.039–1.145)	<0.001	1.101 (1.010–1.199)	0.028	1.061 (0.985–1.143)	0.119

cAIx: carotid augmentation index; CI: confidence interval; cf-PWV: carotid–femoral pulse wave velocity; cPP: carotid pulse pressure; HR: hazard ratio; MACE: major adverse cardiac events, including re-hospitalization for heart failure, non-fatal myocardial infarction, non-fatal stroke, and death; Pb: amplitude of the backward pressure wave.

## Discussion

Due to the limited evidence of treatment, HFmrEF was recently classified as the transition between HFrEF and HFpEF [30]. The present study demonstrated that the clinical and echocardiographic characteristics of HFmrEF, including age, gender, co-morbidities, and left ventricular geometry and functions were usually the intermediates between HFrEF and HFpEF. Given the patients may experience comparable improvements in cardiac performance and vascular resistance after the acute management, arterial stiffness and wave reflection would predominantly present in patients with HFmrEF, comparing to HFrEF. During the index hospitalization, wave reflection phenomenon was significantly obliterated in patients with HFmrEF rather than in the others, by showing the decrease of carotid PP and Pb. Furthermore, HFmrEF and HFrEF would share similar risks for long-term mortality when patients with HFpEF would have better outcomes. When arterial stiffness was related to the prognosis of patients with HFpEF, wave reflection phenomenon correlated with the post-discharge adverse events in patients with HFmrEF and HFrEF.

### The characteristics of myocardial performance in heart failure

It has been noticed that the pathological defect of HFrEF is primarily the myocardial damage, and HFpEF was related to the increased afterload and the subsequent ventricular stiffness [31]. The progressively ventricular-arterial (VA) uncoupling from HFpEF to HFrEF resulted in the geometric changes of the left ventricle that HFrEF would have a dilated ventricle and eccentric hypertrophy while HFpEF usually presented with concentric hypertrophy [12, 32]. Although the left ventricular size of HFmrEF was the intermediate between HFrEF and HFpEF, the present study showed that HFmrEF may have similar geometric changes of eccentric hypertrophy as HFrEF. The results may support that the patients with HFmrEF may have myocardial damage to a certain extend.

### The recovery of pulsatile hemodynamics in acute heart failure

We previous have suggested the excessive wave reflections could have initiated the acute decompensation of heart failure, in addition to volume overload [19, 33]. And the suboptimal recovery of the pulsatile hemodynamics at discharge may indicate incomplete treatment, which was related to adverse clinical outcomes [20]. It is proposed that pulsatile hemodynamics will be much more relevant in patients with HFpEF when they have preserved LV contractility. In patients with HFrEF, the myocardial dysfunction outweighed arterial compliance in the prediction of adverse events. However, the influences of pulsatile hemodynamics in patients with HFmrEF haven’t been elucidated.

All of the three phenotypes of HF in the study presented with typical hemodynamic changes of a rising stroke volume and a decreasing SVRI after the management for AHF [34]. When the increased arterial stiffness and wave reflections were usually the fundamental pathophysiology leading to HFpEF rather than HFrEF [35, 36], HFmrEF unexpectedly exhibited the highest carotid PP, cf-PWV, cAIx and Pb on admission. During the hospitalization, each HF subgroup would experience similar reduction of arterial stiffness due to the shift of the working pressure to a more compliant region by vasodilatory therapy [37]. However, only HFmrEF may encounter a more prominent obliteration of wave reflection than the others after treatment. Although each phenotype of HF was characterized by various risk factors, including age, morbidities and renal functions, which may confound the measures of pulsatile hemodynamics. In cohort A, we analyzed hemodynamic changes in each individual, which was independent of baseline characteristics. In short, the study results may indicate that the acute perturbation of wave reflection phenomenon involves the decompensation of HFmrEF.

### The clinical outcome of each phenotypic heart failure

It was suggested a threshold effect of LVEF on the prognosis of CHF in MAGGIC study that the linear association between LVEF and mortality may no longer exist in the patients with LVEF of ≥40% [17]. The findings may support that HFmrEF would have a better survival rate than HFrEF. However, Solomon et al. reported there was no survival discrepancy regardless of LVEF among the CHF patients with LVEF of ≥45% in CHARM study [14]. In the present study, we demonstrated in AHF patients that HFmrEF shared similar mortality risks as HFrEF when HFpEF had better survival. The risks of mortality remained high in HFmrEF after accounting for age, sex, renal function and hemoglobin. The results may imply a need for evidence-guided therapies in the management of HFmrEF.

### Pulsatile hemodynamics and clinical outcomes in each phenotypic heart failure

Arterial stiffness and wave reflection has been associated with myocardial performance and possibly increase the incident heart failure [38–41]. The present study also supported that cf-PWV was associated with the post-discharge adverse events in patients with HFpEF. In contrast, carotid PP and Pb were correlated with clinical outcomes in subjects with HFrEF. In patients with HFmrEF, only Pb was predictive of adverse events. The study results may support the wave reflection phenomenon a major prognostic indicator in HFmrEF.

## Conclusion

Among patients hospitalized for AHF, those with HFmrEF may have clinical and echocardiographic characteristics intermediates between HFrEF and HFpEF. However, HFmrEF would have left ventricular geometric changes as HFrEF, when both of them presented more eccentric hypertrophy than HFpEF. In addition, subjects with HFmrEF were characterized with increased pulsatile hemodynamics, including PP, arterial stiffness and wave reflection. The impaired LV function coupled with enhanced pulsatile hemodynamics may suggest the unfavorable ventriculo-arterial coupling in HFmrEF. Therefore, HFmrEF would have worse clinical outcomes than HFpEF. The reduction of wave reflection was significant in those with HFmrEF and the pre-discharge level of wave reflection, such as Pb, was associated with adverse events. Given wave reflection predominates the prognosis of HFmrEF, future study is needed to develop the tailored therapy for the specific phenotype of HF.

### Study limitations

There were several limitations of this study. First, we have conducted delicate studies to demonstrate the hemodynamic features of various phenotypic HF. Given the population of Cohort A was relatively small, the statistical might not be sufficient to demonstrate some small discrepancies, if any. In addition, the subjects of Cohort A were of sinus rhythm. Therefore, the study results of pulsatile hemodynamics may only be cautious generalized to other population. Second, the study population was enrolled as their first visit to our hospital for AHF. However, majority of them have encountered the decompensations rather than de novo events. For that reason, future studies with the enrollment of de novo HF are needed to figure out the longitudinal outcomes of HFmrEF. Third, the LVEF were conducted by Simpson’s method and M-mode modified Ellipsoid method in cohort A and B, respectively. We measured the inter-rater reliability for HF phenotypes in 18307 subjects in whom both LVEF data were obtained from January 2014 to December 2015. The Cohen's kappa coefficient was 0.607, represented the substantial agreement of HF phenotypes from two methods. (p value < 0.001)

## Supporting information

S1 Table The pulsatile hemodynamics of the study population. (DOCX)

S2 Table Comparison of the baseline characteristics between Cohort A and B. (DOCX)
