# Supplementary material for: Perturbations of pulsatile hemodynamics and clinical outcomes in patients with acute heart failure and reduced, mid-range or preserved ejection fraction
Source: PLoS One. 2019 Aug 5;14(8):e0220183. doi: 10.1371/journal.pone.0220183 (PMC6681962; doi:10.1371/journal.pone.0220183)
Supplement: S1 Table — (DOCX) [file pone.0220183.s001.docx]

**S1 Table. The pulsatile hemodynamics of the study population**

|  | | | | |
| --- | --- | --- | --- | --- |
|  | **HFrEF (n=138)** | **HFmrEF (n=36)** | **HFpEF (n=56)** | **P value** |
| ***Pulsatile hemodynamics, on admission*** | | | | |
| SV (ml) | 39.0 ± 13.9 | 44.0 ± 14.2 | 52.3 ± 15.2 | <0.001 |
| SVRI (dyn*s/cm5*m2) | 3572 ± 1276 | 3713 ± 1313 | 3252 ± 1020 | 0.172 |
| cPP (mmHg) | 40.1 ± 12.4 | 52.3 ± 19.0 | 50.7 ± 13.1 | <0.001 |
| cf-PWV (m/s) | 12.9 ± 5.0 | 15.2 ± 4.8 | 13.7 ± 4.9 | 0.053 |
| ba-PWV (m/s) | 13.0 ± 3.2 | 14.0 ± 3.0 | 13.2 ± 3.3 | 0.295 |
| cAIx (%) | 11.8 ± 22.3 | 18.6 ± 18.2 | 12.7 ± 22.6 | 0.254 |
| Pf (mmHg) | 33.5 ± 10.8 | 40.4 ± 12.5 | 42.3 ± 10.8 | <0.001 |
| Pb (mmHg) | 14.4 ± 4.7 | 19.4 ± 8.0 | 17.8 ± | <0.001 |
| ***Pulsatile hemodynamics, pre-discharge*** | | | | |
| SV (ml) | 42.4 ± 14.2 | 47.6 ± 15.4 | 54.3 ± 15.5 | <0.001 |
| SVRI (dyn*s/cm5*m2) | 3232 ± 1170 | 3382 ± 1074 | 3150 ± 1016 | 0.629 |
| cPP (mmHg) | 39.9 ± 11.7 | 47.9 ± 14.6 | 49.4 ± 12.2 | <0.001 |
| cf-PWV (m/s) | 11.8 ± 4.2 | 13.6 ± 4.3 | 13.1 ± 4.7 | 0.032 |
| ba-PWV (m/s) | 13.3 ± 3.8 | 13.1 ± 2.6 | 13.5 ± 3.6 | 0.839 |
| cAIx (%) | 15.9 ± 21.7 | 22.5 ± 14.2 | 18.9 ± 15.5 | 0.162 |
| Pf (mmHg) | 31.1 ± 8.1 | 35.5 ± 9.7 | 38.6 ± 9.1 | <0.001 |
| Pb (mmHg) | 14.8 ± 5.0 | 17.8 ± 5.8 | 18.1 ± 5.2 | <0.001 |
| ***Pulsatile hemodynamics, change between admission and discharge*** | | | | |
| SV (ml) | 3.33 ± 10.2 | 3.86 ± 12.4 | 2.01 ± 11.5 | 0.067 |
| SVRI (dyn*s/cm5*m2) | -322 ± 1064 | -238 ± 1330 | -80 ± 1115 | 0.422 |
| cPP (mmHg) | -0.2 ± 2.9 | -4.4 ± 9.1 | -1.3 ± 12.3 | 0.090 |
| cf-PWV (m/s) | -1.1 ± 2.9 | -1.5 ± 2.5 | -0.6 ± 2.7 | 0.235 |
| ba-PWV (m/s) | 0.23 ± 2.60 | -0.86 ± 3.02 | 0.16 ± 2.64 | 0.077 |
| cAIx (%) | 4.0 ± 16.4 | 3.9 ± 11.5 | 6.2 ± 19.7 | 0.695 |
| Pf (mmHg) | -2.4 ± 8.3 | -4.9 ± 7.5 | -3.8 ± 11.3 | 0.272 |
| Pb (mmHg) | 0.4 ± 3.9 | -1.6 ± 4.1 | 0.1 ± 4.7 | 0.035 |

ba-PWV: Brachial-ankle pulse wave velocity; cAIx: carotid augmentation index; CI: confidence interval; cf-PWV: carotid–femoral pulse wave velocity; cPP: carotid pulse pressure; Pb: amplitude of the backward pressure wave; Pf: amplitude of the forward pressure wave.
