# Supplementary material for: Perturbations of pulsatile hemodynamics and clinical outcomes in patients with acute heart failure and reduced, mid-range or preserved ejection fraction
Source: PLoS One. 2019 Aug 5;14(8):e0220183. doi: 10.1371/journal.pone.0220183 (PMC6681962; doi:10.1371/journal.pone.0220183)
Supplement: S2 Table — (DOCX) [file pone.0220183.s002.docx]

**S2 Table Comparison of the baseline characteristics between Cohort A and B**

|  | **Cohort A**  **(n=230)** | **Cohort B**  **(n=2667)** | **P value** |
| --- | --- | --- | --- |
| ***Age (years)*** | 69.8 ± 15.6 | 76.3 ± 12.9 | <0.001 |
| ***Male gender, n (%)*** | 179 (78.2) | 1783 (66.8) | <0.001 |
| ***1-year Mortalities, n (%)*** | 46 (20.0) | 567 (21.2) | 0.367 |
| ***Co-morbidity, n (%)*** |  |  |  |
| HFrEF | 138 (60.0) | 685 (25.6) | <0.001 |
| Hypertension | 92 (67.2) | 28 (77.8) | <0.001 |
| Diabetes mellitus | 109 (47.6) | 997 (37.3) | 0.001 |
| Coronary artery disease | 133 (57.8) | 949 (35.5) | <0.001 |
| Dyslipidemia | 57 (25.3) | 270 (10.1) | <0.001 |
| ***Echocardiography*** |  |  |  |
| LVEF (%) | 37.8 ± 15.1 | 54.5 ± 18.8 | <0.001 |
| Septal E/e’ | 19.0 ± 9.9 | 17.9 ± 7.9 | <0.001 |
| LA diameter (mm) | 41.3 ± 6.4 | 45.7 ± 8.8 | <0.001 |
| LVIDd (mm) | 59.2 ± 10.2 | 54.4 ± 10.1 | 0.773 |
| LVIDs (mm) | 46.8 ± 11.9 | 38.7 ± 12.2 | 0.217 |
| Eccentric hypertrophy, *n (%)* | 69 (30.0) | 492 (18.4) | <0.001 |
| ***Hemogram and Biochemistry, on Admission*** | | | |
| Hemoglobin (g/dl) | 12.0 ± 2.2 | 11.7 ± 2.2 | 0.829 |
| eGFR (mL/min/1.73m^2^) | 52.6 ± 27.8 | 52.9 ± 30.3 | 0.100 |
| Sodium (mEq/L) | 138.3 ± 4.6 | 138.8 ± 4.7 | 0.580 |
| Potassium (mEq/L) | 4.08 ± 0.65 | 4.10 ± 0.69 | 0.974 |
| * Ln NT-proBNP (pg/ml) | 7.72 ± 1.57 | 8.58 ± 1.37 | 0.022 |
| ***Medications, n (%)*** |  |  |  |
| Beta-blocker | 140 (62.2) | 1703 (63.7) | 0.350 |
| RAS inhibitor | 72.7 (58.7) | 2239 (83.8) | <0.001 |
| Spironolactone | 132 (58.7) | 1550 (58.0) | 0.453 |
| Digoxin | 44 (19.6) | 915 (34.2) | <0.001 |

*Geometric means and standard deviation

e’: early diastolic tissue velocity mitral annulus; E/e’: ratio of early ventricular filling velocity (E) to early diastolic tissue velocity mitral annulus; EF: ejection fraction; eGFR: estimated glomerular filtration rate; LA diameter: the diameter of left atrium; HFrEF: failure with reduced ejection fraction; LV: left ventricular; LVIDd: left ventricular internal diameter at end diastole ; LVIDs: left ventricular internal diameter at end systole ; NT-proBNP: N-terminal pro-brain natriuretic peptide; RAS inhibitor: renin-angiotensin system inhibitor
